# Is the number of procedures completed a valid indicator of final year student competency in operative dentistry?

**DOI:** 10.1038/s41415-021-2967-2

**Published:** 2021-05-28

**Authors:** Luke J. Dawson, Kathryn Fox, Mark Jellicoe, Elliot Adderton, Vince Bissell, Callum C. Youngson

**Affiliations:** School of Dentistry, University of Liverpool, Pembroke Place, Liverpool, L3 5PS, UK

## Abstract

**Introduction** Concerns regarding the preparedness of dental graduates often focus on the number of procedures undertaken before qualification.

**Aims** To assess the validity of numerical requirements in determining whether a student has sufficient competency to graduate.

**Materials and methods ** In total, 50,821 observations of student activity while on the restorative clinic, for two integrated student cohorts (n = 139), were continuously recorded using the LiftUpp system from their third year to graduation. Analysis of data determined the most frequent type, location and difficulty of the direct restorations provided.

**Results ** Four groups of students, categorised by the total number of restorations they provided, were identified. Those with the most restorations (70+) had a statistically significantly greater experience in restoring different tooth surfaces, in different quadrants, on different patients than those with the least (40-49). However, increased numbers did not correlate with increasing difficulty, suggesting that students simply became more adept at easier procedures.

**Discussion ** Using a derived measure of 'consistency', it was observed that increased performance consistency was not correlated with greater overall experience. Furthermore, only 4% of students were 100% consistent in their performance in their final year.

**Conclusions ** These data suggest that a 'numerical requirements' approach cannot be used, in isolation, as a valid measure of the competency of an individual. Further work is needed to validate consistency as a holistic measurement to characterise the performance of graduates.

**Supplementary Information:**

Zusatzmaterial online: Zu diesem Beitrag sind unter 10.1038/s41415-021-2967-2 für autorisierte Leser zusätzliche Dateien abrufbar.

## Key points

- The numerical requirement approach alone should not be used to make valid judgements over the competency of a dental student to undertake direct restorations.
- Consistency as a holistic measure of performance may be more suitable to assess the set of skills required.
- Students are not usually 100% consistent in their performance by graduation.

**Supplementary Information:**

Zusatzmaterial online: Zu diesem Beitrag sind unter 10.1038/s41415-021-2967-2 für autorisierte Leser zusätzliche Dateien abrufbar.

## Introduction

From childhood onwards, we receive the consistent message that *'*practice makes perfect'. Consequently, we tend to make the natural assumption that there is a direct relationship between the level of experience gained and the performance achieved*.* Therefore, it would not be surprising if the view persisted that experience (the number of procedures completed) can serve as a proxy for clinical competency, on the basis that after a certain exposure, *'*it [competency] can be expected in most cases*'*.^1^ It is of note that a recent report from the General Dental Council (GDC) has revealed concern among dental foundation training educational supervisors about preparedness for practice of new graduates, a situation that has been equated to a lack of clinical experience in undergraduate training.^2^ While we accept it is likely that many undergraduate programmes no longer use experience alone as a measure of preparedness, a concern over numerical experience is often present, in the background, when student progression decisions are being made. Therefore, it is important to investigate the extent to which numerical experience data can be used to validly inform decisions over competence.

Validity is the ability of a tool to measure the variable that it claims to target.^3^ Validation of any assessment method also depends upon questioning the interpretation of the data gathered. Crucially, if the data are found to be untrustworthy, it undermines the conclusions that can be drawn, rendering them meaningless.^4^^,^^5^ This is a key point, as it would be illogical to base a decision upon the premise that 'using something is better than nothing' when the 'something' is inherently flawed.

In the current study, we have employed a data-driven methodology to examine the validity of the numerical experience approach to measuring competence. Specifically, we explored whether the (traditionally accepted) data describing the number of procedures performed by a student to the required standard (what we have called a 'count: quality' strategy) is an appropriate approach to determine competence. Direct restorations were selected as the basis for the analysis, due to the large amounts of data that were available for each student, as well as recognising that this represents one of the fundamental technical skills in dentistry.

The processes behind validation have moved increasingly to argument-based iterative approaches.^6^^,^^7^ Kane^6^ refers to the claims made for the intended interpretation and use of an assessment as the interpretation use argument (IUA)*.* Validation is the process of establishing the plausibility of the IUA and an important consequence of this is that the evidence required to support the validity argument is proportional to the stakes of the decision being made. This is analogous to the 'burden of proof' in law where criminal cases require proof beyond reasonable doubt, whereas the civil standard works 'on the balance of probabilities'. To support the IUA, Kane^6^^,^^7^ presents a framework that provides a useful approach to testing the validity of the data collected by a 'count: quality' approach.

The first stage of developing an IUA for a 'count: quality' approach is to state the purpose behind gathering the data. In this case, the purpose is to determine the clinical competence of final-year dental students, ready for graduation, in the placement of direct restorations. The next stage is to individually consider each of four inferences described by Kane (scoring, generalisation, extrapolation, implication) to validate the IUA.^6^

The scoring inference considers the approach to generating a fair, accurate and reproducible score. Therefore, the data presented would need to be underpinned by evidence of:

- Independent completion of each restoration recorded for each student (rather than those assisted by a supervisor)
- Staff calibration over what constitutes acceptable quality for sign-off against the GDC Preparing for Practice (PfP) outcomes - ('failing to fail' is a recognised problem in dental education)^8^
- The judgements of multiple, rather than individual, staff members
- A robust system for the collection of the data to prevent data loss or false addition or embellishment.

The generalisation inference seeks to establish how the selected item relates to all possible items that could be assessed in the area under investigation. Therefore, the data need to be underpinned by evidence of:

- The sampling strategy for each individual
- An adequate sample size for each individual
- The differing contexts; for example, surfaces, materials, quadrants, patients, difficulty
- Evidence of inter-rater agreement over the decisions being made.

When key aspects of the above evidence have been met, it is possible to be assured that each restoration has been placed by the student to the appropriate quality in the patient recorded. Furthermore, such evidence also provides support for the extrapolation inference (see below) due to the work-based authenticity of the assessment. However, there are some key assumptions of the 'count: quality' approach that require further investigation and we have approached this by testing two hypotheses:

1. Hypothesis 1: completing a set number of restorations demonstrates that all students have similar evidence of experience with respect to undertaking similar amounts of work on all tooth surfaces, in all quadrants, over a broad range of patients and over a similar range of difficulties (the generalisability inference)
2. Hypothesis 2: there is a direct, systematic relationship between improved technical performance and the number of restorations completed; that is, more technical experience results in an improved performance (the extrapolation inference).

## Methods

### Observations and participants

Retrospective analysis of anonymised data, available within the school's (LiftUpp) database of every observation made by staff on all student direct restoration activity, was undertaken. All the data generated were the result of student activity undertaken on adult patients in the restorative clinics within the dental hospital, under the supervision of restorative staff. Two complete datasets were analysed from all the students in two graduated final-year cohorts, gathered while they were undertaking supervised clinical work within each of their last three clinical years, in the University of Liverpool Dental Hospital. University of Liverpool Ethics Committee approval was gained for this investigation (Ref 2462).

The data were recorded by trained and calibrated restorative staff using LiftUpp, a system originally developed^9^^,^^10^ in the school. The pedagogy upon which this system is based has been published previously^11^ and is grounded in programmatic assessment theory.^12^^,^^13^

The school has no stipulated minimum requirement numbers for the students to progress to their final examinations, but protocols require that data are collected continuously on every clinic, every day, for every student and procedure by a single staff member on each occasion. This data collection strategy aims to avoid Hawthorne effects^14^ and ensures that the resultant data reflect the full developmental profile of each student. In addition, students are rotated between as many different staff as possible over the programme to reduce the risk of bias and to strengthen any decisions made through demonstrating data saturation. Within LiftUpp, observations are made against a six-point developmental indicator (DI) scale (Table 1) that is based on learner independence.^11^

**Table 1:** LiftUpp developmental indicators and descriptions

Developmental indicator	Description
1	Unable to do this. Has caused harm or does not seek essential guidance
2	Unable to do this independently at present. Largely demonstrated by tutor
3	Unable to do this independently at present but able to complete, to the required quality, with significant help, either procedural or by instruction
4	Able to do this partially independently at the required quality, but requires minor help with aspects of the skill, either procedural or through discussion
5	Able to do this independently at the required quality. This may include confirmatory advice from the tutor where the student seeks appropriate assurance
6	Able to meet the outcome independently, exceeding the required quality

For the purposes of this study, a DI of 5 was used as the reference threshold, as this represents 'independence', which is the aim for the end of training when undertaking a longitudinal evaluation of performance.^15^^,^^16^

In common with all procedures using LiftUpp, each staff member records their judgements on an iPad while the procedure is being directly observed. These judgements are made using the DI scale against a set of criterion-referenced questions contained within a specific 'procedure' section. In addition, the system also collects information about the context of the restoration (for example, quadrant, tooth, surface, material) as well as demographic information about the patient under treatment and degree of procedural difficulty. Each stage of the procedure is required to be at an appropriate DI level for the restoration to be deemed successful. This enables the provision of specific feedback^17^ and is intended to drive deliberate practice^18^ in the students. Staff calibration is undertaken within the department as the software allows comparison of the awarded DIs between staff, to identify 'hawks' and 'doves'. Staff receive personalised feedback and continued monitoring of their subsequent practice is undertaken. The rotation of students among all staff and the large number of datasets gathered also tends to normalise for any minor discrepancies in staff calibration.^13^

### Data handling

Anonymised data for direct restorations were gathered from the database using relevant structured query language queries. Statistical analysis was performed using Microsoft Excel and R.^19^ Cohort datasets were compared using a Kolmogorov-Smirnov (K-S) test to ensure that the two separate cohort datasets had the same distribution.

The relationship between the number of restorations completed and the relative experience in relation to quadrants, teeth, tooth surfaces and number of patients managed was established. Self-evidently, due to individual patient treatment needs, it is not possible to provide a uniform experience for all students with respect to which tooth surfaces or quadrants require restoration. However, both of these parameters were taken into account in our calculation of difficulty. The relative level of difficulty of the restorations performed by each student was calculated by establishing the number of 'difficult' restorations and then expressing these as a percentage of the total restorations placed. A restoration was considered relatively more difficult if it was approximal or undertaken on an upper posterior tooth (quadrant 1 or 2, teeth 4-8). In addition, a restoration was also counted as 'difficult' if it had been tagged as 'difficult' by the supervising member of staff for another reason (for example, lack of patient cooperation) at the time of observation.

Although it is accepted that within a numbers-based 'count: quality' approach there is an intrinsic measure of performance (that is, quality), it is not possible to establish how that performance develops over time where the system solely reports the total number of restorations placed at the required quality. Therefore, to investigate changes in performance, we propose a new measure termed 'consistency' (C). Consistency aims to establish the overall longitudinal performance consistency and is calculated using the following formula: C = sum of the lowest DI per restoration (at or above threshold)/total number of restorations.

To illustrate, if the lowest DI for any stage is ≥5, then the whole restoration is returned as a '1'; that is, has been placed independently. Alternatively, if any stage DI is <5, then the restoration is returned as a '0'; that is, has not been placed independently. Applying this process to all restorations placed in the final year of study, for each student, returns a string of '1s' or '0s' which can be used to calculate their C; for example, if ten restorations had been placed by a student in final year with the lowest DIs being 4, 3, 5, 5, 6, 6, 4, 5, 4, 6, respectively, then this would be returned as 0, 0, 1, 1, 1, 1, 0, 1, 0, 1 with C *=* 6/10; that is, 0.6 or 60%.

Following initial data gathering, to allow further analysis, students were categorised into one of four groups - based on the total number of approximal, incisal edge, smooth surface and/or occlusal surface restorations they had undertaken: group 1, 40-49; group 2, 50-59; group 3, 60-69; and group 4, 70+ (Table 2). However, where restoration experience across both cohorts was less than 40 (n = 4), those data were excluded from group-level analysis.

**Table 2:** Descriptive analysis of group data for all students in both cohorts in relation to tooth surface, number of teeth restored in a quadrant and mean difficulty of all the teeth restored; values are median (min-max) unless otherwise stated

Group (completed restorations; number of students)	1 (40-49; n = 27)	2 (50-59; n = 55)	3 (60-69; n = 27)	4 (>70; n = 26)	Total (n = 139)
**Surface**					
Approximal anterior	7 (2-14)	8 (2-21)	11 (2-17)	13 (6-25)	9 (2-25)
Approximal posterior	12 (4-21)	14 (7-33)	16 (7-29)	16 (6-30)	14 (4-33)
Incisal edge	3 (0-24)	4 (0-31)	9 (0-22)	7 (0-37)	6 (0-37)
Smooth surface	11 (2-19)	12 (2-23)	14 (5-37)	23 (6-43)	13 (2-43)
Occlusal surface	5 (2-11)	8 (2-17)	8 (2-14)	10 (1-21)	7 (0-21)
**Quadrant**					
1	12 (8-22)	15 (3-25)	18 (8-30)	18 (11-38)	15 (3-38)
2	14 (7-23)	15 (6-31)	12 (8-28)	12 (14-37)	16 (4-37)
3	11 (2-16)	12 (3-23)	16 (7-23)	18 (12-28)	13 (2-28)
4	10 (4-16)	12 (4-21)	13 (4-24)	18 (11-27)	12 (3-27)
Difficulty n (mean ± SD)	27.81 (± 6.49)	31.45 (± 6.36)	36.81 (± 7.03)	43.12 (± 8.78)	34.04 (± 8.75)
Difficulty % (mean ± SD)	60.11 (± 12.71)	57.65 (± 11.02)	57.29 (± 10.61)	54.45 (± 10.27)	56.32 (± 11.09)

Note:

Where restoration experience across both cohorts was lower than 40 (n = 4), results were excluded from group-level analysis.

Previous research from UK dental schools noted that the mean number of restorations at graduation would be approximately 50-60,^1^ a range comparable to the current study group 2. Therefore, by exploring individual variation in this representative group in more detail, it is possible to evaluate the amount of variability between individuals who have an 'average level' of undergraduate experience.

## Results

A K-S test of data indicated that there was no significant difference (p >0.05) between the two cohort distributions, which supported the combining of the cohort data before further analysis.

A total of 139 students had observations, with respect to direct restorations placed, recorded by an average of ten (± 3) different restorative staff per student. Overall, 50,821 observations were recorded, with 14,295 taking place in the test cohort's final year of study.

Overall, at a cohort level, the data (Table 2) demonstrate the trend that experience, in terms of range of surfaces restored, quadrants in which restored teeth were situated and the number of patients treated, increases with activity, as might be expected (additional detail and analysis is also provided in the online supplementary information).

### Representative group (group 2): total restorations completed and experience

**Fig. 1:**
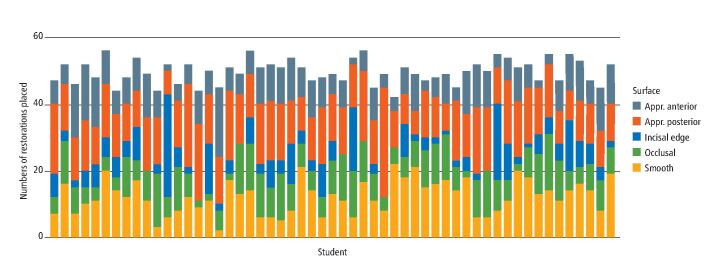
Group 2 (50-59) data showing the relative different tooth surfaces treated

A total of 55 students, who provided 50-59 restorations, constitute group 2. The data in Figure 1 show a high degree of variability between the individual students in terms of experience of restoring different tooth surfaces, despite the fact they have all placed a similar number of restorations (note that only the five most common types of restorations are shown). These differences in experience can be quite large. For example, with respect to incisal edge restorations, the minimum experience within this group is 0 and the maximum is 23. For approximal posterior restorations within this group, the minimum experience is 7 and the maximum is 33.

**Fig. 2:**
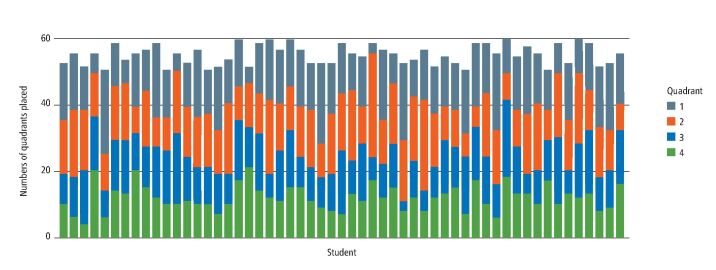
Group 2 (50-59) data showing the numbers of direct restorations placed relative to quadrant

Similar variability was found with respect to working in different quadrants (Fig. 2), with a number of individuals having undertaken six or less restorations in Q1, while others have comparatively little experience in the lower arch (Q3 and Q4).

**Fig. 3:**
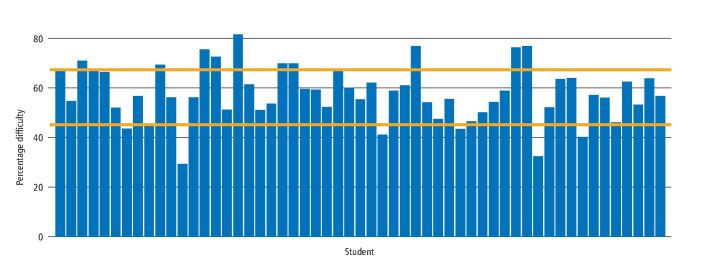
Relative difficulty of the restorations placed by learner within group 2 (50-59 direct restorations). Yellow horizontal lines indicate ± 1 SD of the combined cohort mean

Figure 3 shows the variability in the difficulty of procedures undertaken within group 2. The data show a wide variation in the percentage of difficult restorations undertaken by students, with a range between 29% and 81%.

### Consistency and differentiation across all groups

**Fig. 4:**
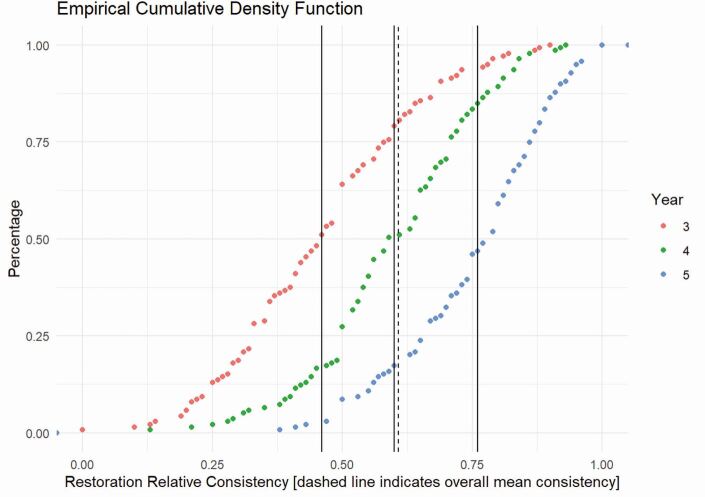
Shows the cumulative frequency of percentage of students at each level of consistency while in each year of study. The combined cohort mean consistency for each year of study is shown, along with the overall combined cohort mean consistency of 0.59 (dotted line)

**Fig. 5:**
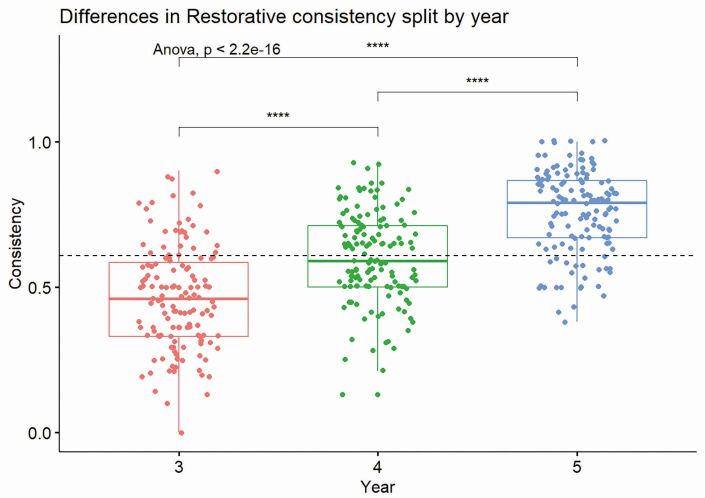
Shows a box and whisker plot of performance consistency for individual students, as they progress through the years of study. The box represents the mean and interquartile range, and the whiskers the minimum and maximum consistencies. The dotted line represents the overall cohort mean consistency of 0.59

Figure 4 uses a cumulative density function to explore 'consistency' in the placement of direct restorations per student, per year of study. The data show that it is possible to differentiate between both students and years of study. Mean consistency scores, illustrated in Table 3, indicate a linear increase from third to fourth and then on to fifth year. However, these data also demonstrate that at the end of fifth year, only 4% (6/139) of students had been 100% consistent in their ability to place direct restorations independently (Figures 4 and 5).

**Table 3:** Performance consistency and the median number of restorations by year of study

Year	Mean consistency (± SD)	Restorations median
3	0.46 (± 0.18)	16
4	0.60 (± 0.16)	24
5	0.76 (± 0.14)	16
All	-	56

Supporting the linear relationship between the years of study, analysis of variance (ANOVA) revealed a significant effect of year of study on overall consistency (p <0.001). Bonferroni *post-hoc* comparisons indicated significant differences between third and fourth year, indicating a 14% increase in consistency between the years (p <0.001). Furthermore, a significant (p <0.001) difference of 15% between fourth- and fifth-year performance consistency was observed. This was associated with an overall 29% improvement in performance consistency between third and fifth year (p <0.001) (Table 3, Figure 5).

### Relationship of consistency to the total teeth restored

**Fig. 6:**
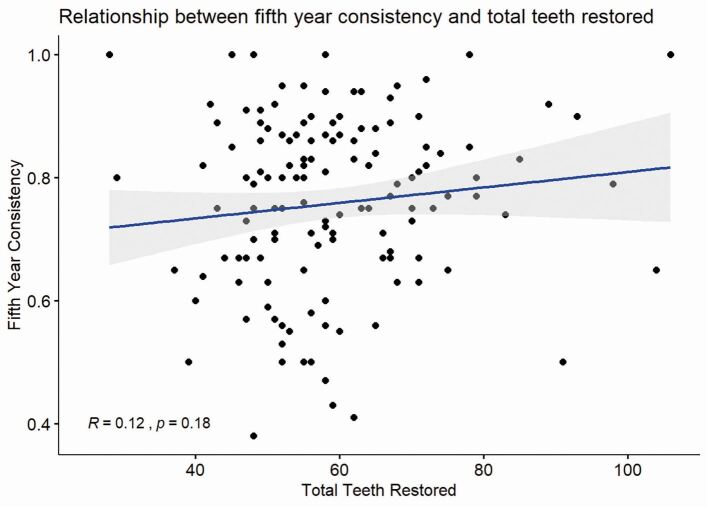
Scatterplot showing the relationship between the total teeth restored and the overall consistency in the fifth year of study. The line represents the calculated linear regression line

Correlation analysis found a weak, non-significant relationship between the total number of teeth restored and fifth-year consistency - r = 0.12 (p = 0.18) - shown in Figure 6.

## Discussion

To ensure the public receives safe and effective care from new graduates, it is essential^20^ to have demonstrably valid evidence to ensure that the learning outcomes within PfP^11^^,^^21^ have been met.

Using Kane's validity framework^6^ and direct restorations as a model test system, we analysed a large volume of data in order to investigate the validity of using experience, in terms of numbers of procedures completed, as a proxy for competence. In particular, we tested two hypotheses: 1) completing a set number of restorations demonstrates that all students have similar evidence of experience; and 2) there is a direct, systematic relationship between improved technical performance and the number of restorations completed.

### The numerical relationship between restorations completed and experience

The finding that increasing the total number of restorations also increases the experience of working in differing contexts (Table 2; Figures 1, 2 and 3; and online Supplementary Figures 1, 2, 3, 4 and 5) is in keeping with hypothesis 1. However, it is also of note that as the total restoration provision increases, the data demonstrate a statistically significant increase in the more straightforward, and less time-consuming, incisal edge and smooth surface restorations (Table 2, Figure 1). Furthermore, the cohort difficulty data also indicate a downward trend as total restorations increase (Table 1). Taken together, these data strongly suggest that some students are adept at providing simple restorations on compliant patients, but do not necessarily expand their skillset in complex cases. In this case, 'more' is not always 'better' and this suggests that systems that reward minimum numbers are likely to provide the wrong educational impact.^11^ Realistically, a large number of restorations could reflect only very simple procedures undertaken on easily accessible teeth.

To further explore hypothesis 1**, **we determined that a total of 50-59 direct restorations has previously been identified as an average range for many students across the UK^1^ and this matches group 2 of our study. However, the ability to satisfy learning outcomes is not done at the cohort, but at the individual, level. Consequently, variability between individuals is an important consideration and so we evaluated the individual variation in group 2 (Table 2).

The data for tooth surfaces and quadrants (Figures 1 and 2) showed a wide individual variation. The educational significance of this is uncertain but it does suggest that total numbers of restorations completed reveal far from the whole story and that much more granular information is required if the true range of a student's experience is to be understood.

To further investigate differing experience, we consolidated the variables of tooth surfaces, quadrants and external factors into a single estimate of overall difficulty of the teeth managed by each student, as understanding difficulty is a fundamental requisite of good assessment.^22^ Part of our rationale to include tooth surfaces in this category was that approximal restorations have been a staple of dental assessment (for example, 'the class II test'), due to these being considered more difficult, and the restoration of upper posterior teeth is more likely to involve the use of indirect vision.

The data in Figure 3 show the variation in overall difficulty (the number of difficult restorations expressed as a percentage of the total placed) for those individuals in group 2. Using the total combined cohort difficulty mean as a reference value, it can be seen that 17 students are just above, or below, one standard deviation of the total cohort mean. The educational impact of this is potentially significant because being appropriately challenged is fundamental to improving performance^18^^,^^23^ as well as helping to develop self-efficacy, goal setting^24^ and self-regulation.^25^ Furthermore, it is well known that students can demonstrate a 'fixed mindset'^26^ and, as such, suffer with a fear of failure^27^^,^^28^ and consequently avoid challenge. Therefore, the finding that 15% of group 2 are working at, or below, the lower end of difficulty implies that they may be demonstrating these behaviours. In addition, a lower level of difficulty would likely also be associated with a false increase in the perceived quantity and quality of the performances if this was recorded using a 'count: quality' approach, especially in the later years of study.

Taken together, these data suggest that hypothesis 1(completing a set number of restorations demonstrates that all students have similar evidence of experience) should be rejected, as data collected from a 'count: quality' approach cannot support the conclusion that providing a set number of restorations reflects equivalent experience for all students. Furthermore, the data suggest that increasing the number of procedures completed may not be the answer unless the context of the increased experience (for instance, the difficulty of the additional procedures undertaken) can be controlled.

### Consideration of the use of consistency

The data shown in Figures 4 and 5 provide evidence to support the validity of the consistency measure because they demonstrate it is capable of differentiating between individual students and between their years of study. In addition, data from a recent study exploring the generalisability inference, which also used big data approaches to work-based assessments, demonstrated high levels of reliability.^29^ While we accept that further evidence with respect to generalisability and understanding the sources of variance within the consistency measure is needed to fully validate it, the available evidence suggests that the data gathered using consistency are likely to be suitable to explore the relationship between experience of direct restorations and performance development.

A noteworthy finding, using consistency, was that only 4% of final-year students showed 100% consistency (Figures 4 and 5), a situation that some may find surprising - although we should all recognise that we have 'good' and 'bad' days and few practising clinicians are 100% consistent in all circumstances. It is therefore highly likely that a significant number of students will not be 100% consistent (at the level of independence) by the end of their final year, supporting Chambers' assertion that 'five years is enough time for a good start'.^25^ Further work is now needed to fully understand what a 'good start' (that is, a safe beginner) actually looks like, but it is something that the GDC has also recognised that the profession must actively consider^2^ to prevent potential unrealistic expectations in postgraduate training, as well as to inform progression decisions during undergraduate training. It is likely that a 'safe beginner' graduate would significantly develop both their capabilities^30^ and consistency towards the end of foundation training, with the establishment of full independence (high levels of consistency under all circumstances - an 'expert') sometime later.

### The relationship between technical skill and experience

A major assumption in using data from a 'count: quality' approach is that, after a certain exposure, 'it [competency] can be expected in most cases*'.*^1^ The data shown in Figure 6 suggest that any relationship between total experience and consistency in final-year performance is, at best, very weak. Therefore, these data do not support hypothesis 2 (there is a direct and consistent relationship between improved technical performance and the number of restorations completed).

However, the data did show a potentially important relationship as consistency, in each year, is also related to consistency in later years (although the data do not allow individual inferences to be drawn). These data may suggest that improved performance is not a function of specific experience in a single area (that is, restorations), but rather the outcome of integrated experience across many areas within the programme - a situation that is also likely further supported by longitudinal personal development and growing maturity. Further work is needed to explore this concept that is grounded in the personal development and self-regulated learning approaches associated with a 'growth mindset'.^26^

### The potential of consistency

Consistency also offers some possibilities with respect to informing student development, making valid progression decisions and providing a meaningful transferable dataset to foundation training, which has been an enduring goal for over ten years.^31^

Having a transparent dataset, grounded in performance, may also improve student confidence over discussing their ongoing developmental needs with foundation trainers/educational supervisors, rather than avoiding failure^28^ by claiming that they have not been taught the skill as an undergraduate in the hope that they will not be asked to do it unsupervised. This 'avoidance tactic' is a situation that anecdotal evidence and personal experience suggest may underlie some of the current controversy over a perceived lack of graduates' experience.

The data in Figures 4 and 5 suggest that it is possible to use consistency to develop a growth curve approach which can help inform individual progression decisions. Such an approach would offer reassurance, and evidence, that each individual student had met the learning outcomes without resorting to 'one size fits all'; that is, everyone must have the same amount of experience. Consistency could also be used to explore a student's holistic skillset through nesting related skills, rather than the current approach of looking at a single aspect, akin to the use of entrustability in medical education.^32^^,^^33^

Other grading systems that are widely adopted in dental education may also be able to be used to provide an estimate of consistency, albeit one with perhaps limited transparency and generalisability. For example, where a school uses an 'A, B, C, D' or similar system, the reference threshold could be set as the sum of all restorations at 'A' for all aspects of the restoration and the consistency calculated accordingly. However, for valid inferences to be drawn, Kane's framework^6^ would still need to be fulfilled.

For data gathered using the consistency measure to be meaningful, it will be necessary to control for both activity and difficulty, because if a student performs a few simple restorations well and then stops, they could appear to be 100% consistent. Therefore, information about the level of activity, contexts and a broad portfolio of activities is needed. This latter requirement could be supported with the longitudinal incorporation of compulsory high-quality simulated cases of increasing difficulty, a situation that would also support regular skills maintenance.

## Conclusions

The data derived from our investigations suggest that, from the perspective of argument-based validity, significant caution must be exercised when inferring competence from the volume of experience alone because, in the undergraduate setting, equivalent volumes of experience between students do not reliably indicate an equivalent range of experience with respect to context or difficulty. Therefore, a full understanding of a student's experience requires much more granular information than the total volume of a particular type of procedure. Furthermore, there is only a very weak (and therefore no meaningful) relationship between the volume of experience and consistency of performance (competency) over a given period of time.

We accept that further work is needed to fully validate consistency as an indicator of competence. Nevertheless, we believe that this study has potentially significant implications. Moreover, the conclusions support the need for a transfer of meaningful information between undergraduate and postgraduate training because they highlight the need for a shared understanding of what the data actually demonstrates. In addition, the data also indicate that determining when a student is ready to make the transition to postgraduate training remains complex, but a rigorous approach to validation of any proposed method of decision-making is essential.

## Supplementary Information

Supplementary analysis and Figures S1-S6 (PDF 256KB)
