# Supplementary material for: Is the number of procedures completed a valid indicator of final year student competency in operative dentistry?
Source: Br Dent J. 2021 May 28;230(10):663–70. doi: 10.1038/s41415-021-2967-2 (PMC8163597; doi:10.1038/s41415-021-2967-2)

Supplementary Material

Analysis by tooth surface

This aimed to establish the relationship between the number of teeth restored and the experience of tooth surfaces treated. A significant difference in experience treating variable tooth surfaces was seen with respect to the overall number of teeth treated, $\chi^2(4) = 186.13$, $p < .001$, $\varepsilon^2 = .28$, indicating a large effect. Dwass-Steel-Critchlow-Flinger (DCSF) pairwise comparisons indicated that, with the exception of approximal posterior and smooth surface procedures (which did not differ significantly) all other surfaces differed at a statistically significantly level (*ps* < .001 [except for the comparison between incisal edge and the occlusal surface where $p = .022$]) (Table 3 and Figure S1).

Figure S1

Cohort level analysis of restorations placed and tooth surfaces restored (Mean values are displayed central to SEM error bars; Median values are non-central)

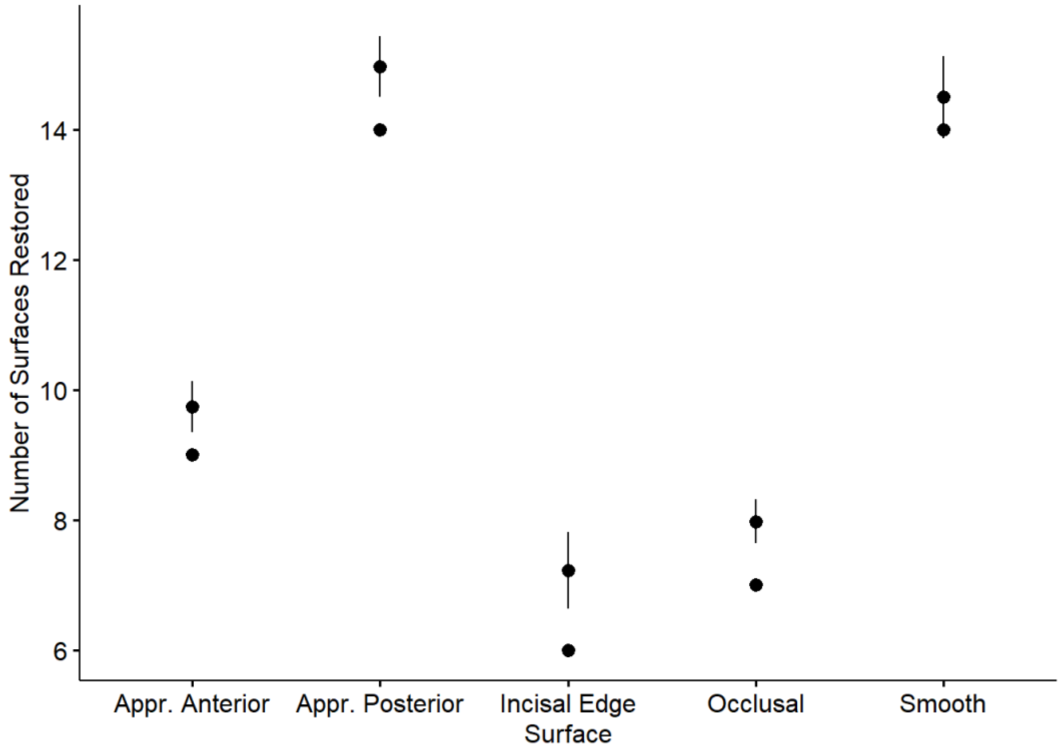

In the next phase, each of the five most common restorative surfaces were examined in terms of Groups to establish how experience differed (Figure S2). In each case, due to the non-parametric nature of the data, a Kruskal-Wallis test examined overall differences and DCSF pairwise comparisons further explored significant main effects.

In relation to anterior approximal surfaces, there was a significant overall effect of group , $\chi^2(3) = 26.07$, p < .001, $\varepsilon^2 = .19$. Learners in Group 4 (70+ restorations) accrued significantly more experience than each of the other Groups (1, $W = 6.31$, $p < .001$; 2, $W = 5.93$, $p < .001$; 3, $W = 3.81$, $p = .035$) groups. There were no statistically significant differences amongst Groups 1-3 in experience of anterior approximal surface restorations.

In relation to the approximal posterior surfaces, there was a significant overall effect of group, , $\chi^2(3) = 9.42$, p = .024, $\varepsilon^2 = .07$. Group 3 (60 – 69) demonstrated a marginal, but still significantly greater experience (W = 3.71, $p = .043$) in this than Group 1 (40 – 49). The remaining groups did not differ statistically significantly from each other in experience of approximal posterior surface restorations, *ps* > .05.

In relation to incisal edges, there was a significant overall effect of group, $\chi^2(3) = 18.89$, $p < .001$, $\varepsilon^2 = .14$. Both the > 70 ($W = 4.08$, $p = .020$) and the 60 – 69 ($W = 4.29$, $p = .013$) learner groups enjoyed significantly greater experience than their colleagues in the 40 - 49 group. Likewise, the 70+ ($W = 4.21$, $p = .015$) and the 60 -69 ($W = 4.56$, $p = .007$) groups enjoyed significantly greater experience than the 50 - 59 group. The 40 - 49 and the 50 -59, and the 60 - 69 and 70+ groups each, did not differ from each other (*ps* > .05).

In relation to occlusal surfaces, there was also a significant overall effect of group experience ($\chi^2(3) = 21.11$, $p < .001$, $\varepsilon^2 = .16$). Each of Groups 4,3 &2 accrued significantly greater experience on the occlusal surface than Group 1 (Gp4, $W = 5.81$, $p < .001$; Gp3, $W = 4.24$, $p = .015$; Gp 2, $W = 5.17$, $p = .001$). Groups 2 and 3 did not differ significantly from each other, and neither did they independently differ from Group 4, *ps* > .05.

Finally, in this phase, a significant effect of learner group was seen on smooth surface experience , $\chi^2(3) = 29.01$, $p < .001$, $\varepsilon^2 = .22$. This was driven by learners in Group 4 who accrued significantly more experience than their counterparts in Group 3 ($W = 4.10$, $p = .019$), in Group 2 (W = 6.60, p < .001) and Group 1 (W = 6.17, p < .001). When comparing all other groups, their experiences did not differ significantly from each other (ps > .05) (Table 1, Figure S2).

Figure S2

Group level analysis of restorations placed and tooth surfaces restored (Mean values are displayed central to SEM error bars; Median values are non-central).

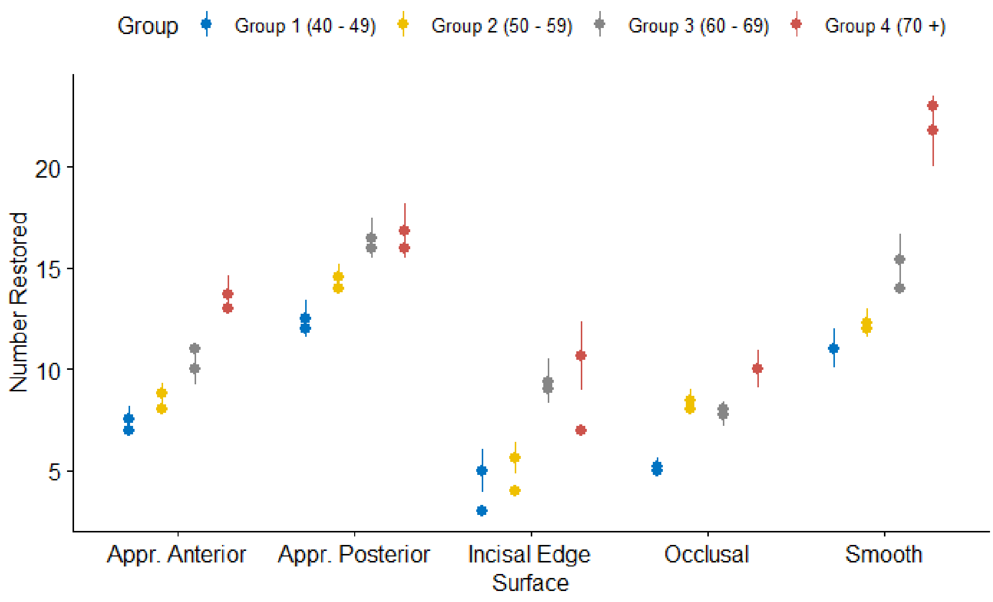

Analysis by quadrant

In this phase of analysis, the upper right quadrant was assigned as Quadrant 1 (Q1), the upper left as Q2, the lower left as Q3 and the lower right as Q4. Again, using Kruskal-Wallis tests, a significant effect of quadrant was reported , χ^2 (3) = 35.10, p < .001, 2 = .07. DSCF pairwise comparisons revealed that, overall, learners were less likely to gain experience in Q3 (W = -4.20, p = .016) and Q4 (W = 6.61, p < .001) than in Q1. Furthermore, overall, learners were significantly less likely to accrue experience in Q3 (W = -4.86, p = .003) and Q4 (W = -7.01, p < .001), than in Q2. Differences between Q1 & 2, and Q3 & 4, were not significant, ps > .05 (Table 3, Figure S3).

Figure S3

Cohort level analysis of restorations placed and quadrants restored (Mean values are displayed central to SEM error bars; Median values are non-central)

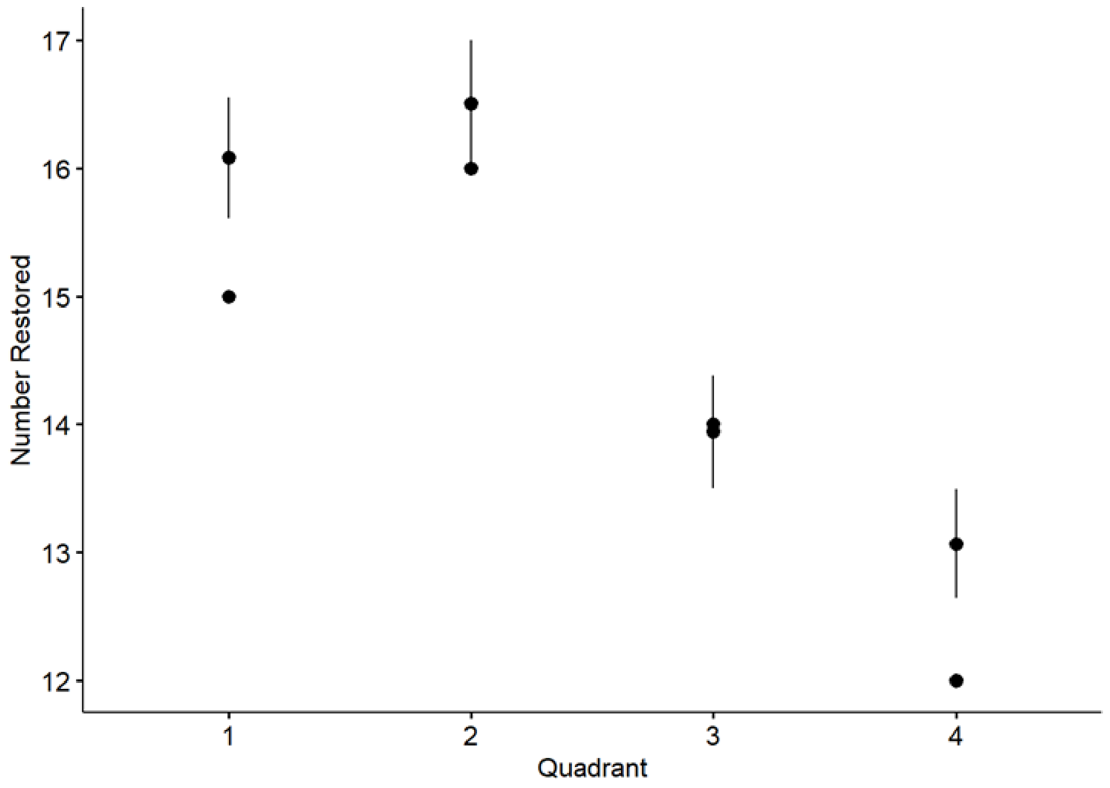

Next, differences by quadrant of the mouth, within the stratified experience groups, was examined (Figure S4). For each quadrant Kruskal-Wallis tests with DSCF pairwise comparison tests were used to examine significant overall effects.

In Q1, a significant effect of experience group was noted, $\chi^2(3) = 29.62$, $p < .001$, $\varepsilon^2 = .22$ Pairwise comparisons revealed that learners in Group 3 ($W = 5.68$, $p < .001$) and Group 4 (W = 6.45, p < .001) accrued significantly greater experience than learners in the Group 1. Also, in Q1 significantly greater experience was gained by Group 3 when compared to Group 2 (W = 3.78, p = .038) and also Group 4 ($W = 5.01$, $p = .002$). However, Groups 3 and 4 did not differ from each other significantly, p > .05.

In Q2, a significant effect of experience group was reported (p <0.001). Pairwise comparisons revealed that the effect of experience in this quadrant was driven by greater experience in Group 4 when compared to each of Group 1 ($W = 7.45$, $p < .001$), Group 2 ($W = 7.20$, $p < .001$), and Group 3 ($W = 5.37$, p < .001). All other groups experience in quadrant two did not differ significantly (*ps* > .05).

In Q3, again, a significant effect of experience group was observed, $\chi^2(3) = 44.38$, $p < .001$, $\varepsilon^2 = .33$. Pairwise comparisons revealed that the effect of experience in this quadrant was driven by significantly greater experience in Group 3 (W = 5.83, p <.001), and Group 4 ($W = 8.08$, $p < .001$), when compared to Group 1. Both Group 3 ($W = 3.83$, $p = .034$) and 4 ($W = 7.00$, $p < .001$) enjoyed significantly greater experience in quadrant two when compared with the Group 2. None of the remaining groups differed from each other in this quadrant (*ps* >.05).

In Q4, a significant effect of experience group was also recorded, $\chi^2(3) = 44.12$, $p < .001$, $\varepsilon^2 =$ .33. Pairwise comparisons revealed that the effect of experience in quadrant four was driven by significantly greater experience in Group 2 ($W = 3.95$, $p = .027$), Group 3 ($W = 4.98$, $p =.002$) and Group 4 ($W = 8.08$, $p < .001$) when compared to Group 1. Group 4 enjoyed greater experience in quadrant four when compared with both Group 3 ($W = 4.14$, $p = 0.18$) and Group 2 ($W = 7.35$, $p < .001$). The Groups 2 and 3 did not significantly differ in experience in quadrant four (p >.05) (Table 3, Figure S4).

Figure S4

Group level analysis of restorations placed and quadrants restored (Mean values are displayed central to SEM error bars; Median values are non-central)

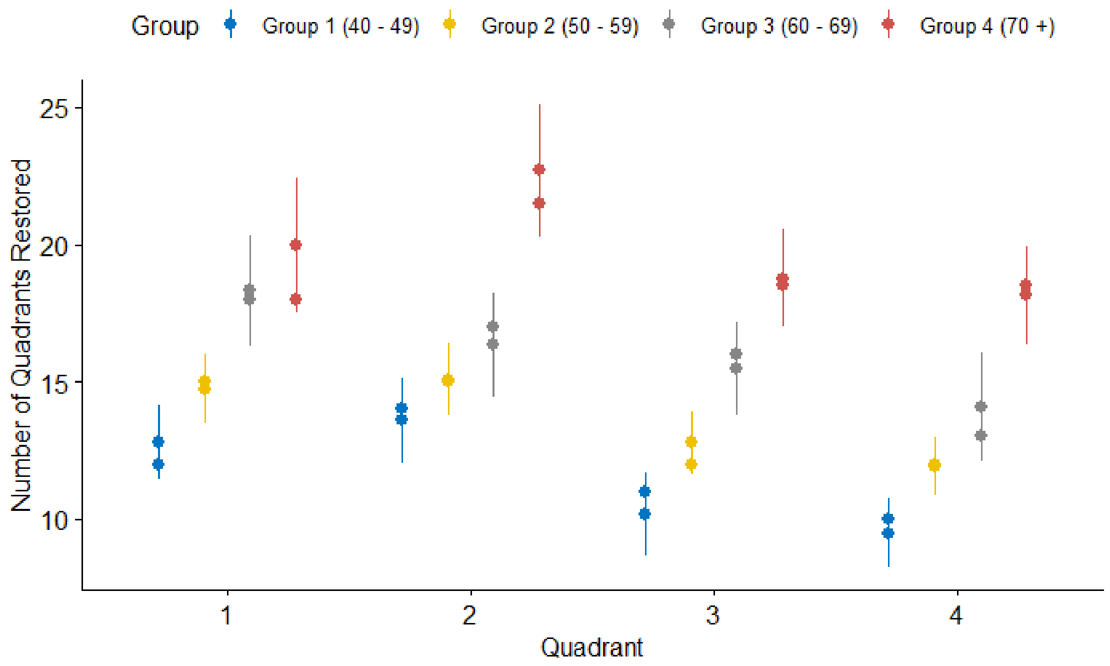

Analysis of relative difficulty

ANOVA was used to investigate relative restoration difficulty between the Groups. Although Figure S5 suggests that difficulty decreases as the learner experience increases, this effect is not significant, $F(3, 131) = 1.14$, $p = .334$, $\eta_p^2 = .03$.

Figure S5

Group level analysis of restorations placed and relative difficulty (Mean values are displayed central to SEM error bars)

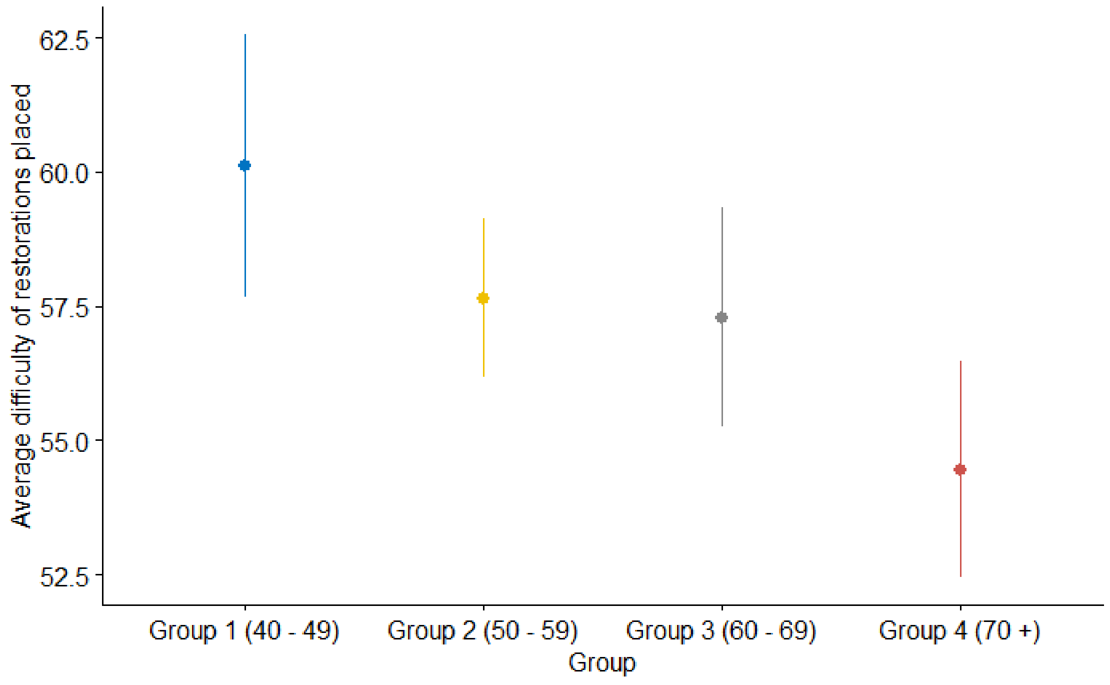

Analysis by unique patients count

ANOVA was also employed to examine whether the number of patients differed between learner experience groups. A significant effect of learner group on the number of patients treated was seen , $F(3, 131) = 4.94$, $p = .003$, $\eta_p^2 = .10$. Bonferroni post-hoc comparisons revealed that this effect was driven by Group 4 examining a greater number of patients than

those in the Group 1 ($p = .002$). This represents a mean difference of 3 patients between the highest and lowest groups. None of the other groups differed from each other significantly (*ps* > .05)(Table 3, Figure S6).

Figure S6

Group level analysis of restorations placed in unique patients (Mean values are displayed central to SEM error bars)

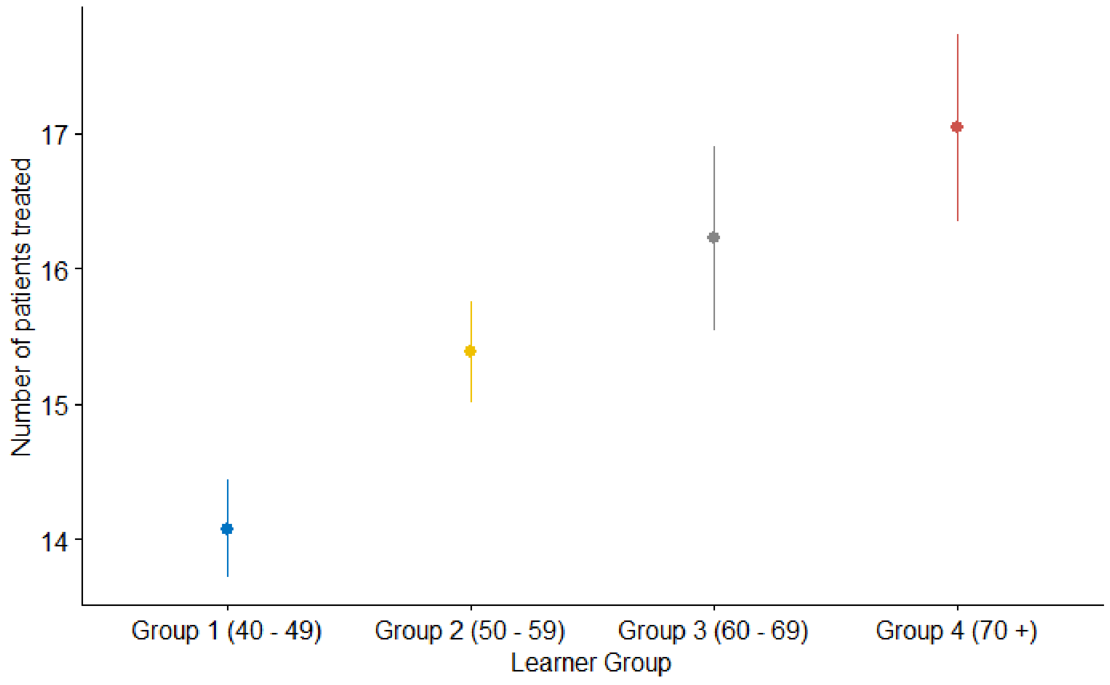

Supplement: Supplementary file 1 — Supplementary analysis and Figures S1-S6 (PDF 256KB) [file 41415_2021_2967_MOESM1_ESM.pdf]
